# Practical Materia Medica for Nurses, with an Appendix

**Published:** 1899-09

**Authors:** 


PRACTICAL MATERIA MEDICA FOR NURSES, with an Appendix. Containing Poisons and their Antidotes, with Poison Emergencies, Mineral Waters, Weights and Measures, etc., etc. By EMILY A. M. STONEY, Graduate of the Training School for Nurses, Lawrence, Mass., etc., etc. Duodecimo, pp. 306. Philadelphia; W. B. Saunders. 1899.

This book is compiled by Emily A. M. Stoney, who a few years ago wrote a very good book for nurses, entitled, Practical points in nursing. This volume contains all that a nurse needs to know concerning the source, action and use of drugs, dosage, and the symptoms and treatment of poisoning. We doubt not that superintendents of training schools will find it an excellent help in the work of the class-room and that graduate nurses who wish some work on materia medica will be pleased with this one. M. J. F.

